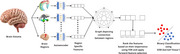# Connecting the Dots with Deep Learning: A Graph‐Based Approach of Alzheimer's Conversion Prediction

**DOI:** 10.1002/alz70856_096798

**Published:** 2025-12-24

**Authors:** Harsh Bhasin, Nishant Rana, Vishal Deshwal, Arush Jasuja, Hardeo Kumar Thakur, Anupama Singh

**Affiliations:** ^1^ Bennett University, Greater Noids, Uttar Prdesh, India; ^2^ Bennett University, Greater Noida, Uttar Pradesh, India; ^3^ Sequoia Insilico Pvt. Ltd., New Delhi, Delhi, India; ^4^ International Centre for Neuromorphic Systems (ICNS), Western Sydney Universiy, Sydney, NSW, Australia; ^5^ New York University, New York, NY, USA

## Abstract

**Background:**

This research aims to improve the prediction of Mild Cognitive Impairment (MCI) conversion to Alzheimer's disease. In order to achieve this, this study focuses on seven specific brain regions identified using the brain atlas. The regions are Hippocampus, Entorhinal cortex, Cerebral cortex, Frontal lobe, Temporal lobe, Parietal lobe, and Occipital lobe. The decay in the gray matter in these regions is associated with the cognitive impairment. This method proposes a novel feature extraction method based on Auto Encoders and then uses these feature to create a graph representing the association between these regions.

**Method:**

The latent representation of the seven regions is found using a novel auto‐encoder based method. This is followed by the formation of a graph, where each of the above regions are nodes and the distance between these nodes is proportional to the inverse of the similarity between the latent representation of the regions.

By examining the relationships between these regions, the study seeks to identify patterns associated with MCI conversion. The method involves flattening the above‐formed graph representation into a 1‐D vector, which serves as a unique feature representation for each brain volume. The classification is done using the Support Vector Machine Linear Kernel and forward feature selection is used for selecting the pertinent features.

**Result:**

The method has been validated using the data has obtained from Alzheimer's Disease Neuroimaging Initiative (ADNI). We collected 75 s‐MRI scans of the patients suffering from MCI who converted to Alzheimer's (MCI‐Converts) and 112 s‐MRI scans of the patients suffering from MCI who did not convert to Alzheimer's (MCI‐Non Converts). The F‐score of the classification is 95.4%, which is better than the state of the art.

**Conclusion:**

The proposed method provides region‐specific insights**,** that is it allows for the identification of specific brain regions that are crucial in predicting MCI conversion. It also opens the doors for network analysis of the connections between regions and provides valuable information about the underlying networks involved in MCI conversion. Furthermore, the method also gives promising results and is more generalizable.